# Characteristics and treatment outcomes of HIV infected elderly patients enrolled in Kisii Teaching and Referral Hospital, Kenya

**DOI:** 10.4314/ahs.v20i4.6

**Published:** 2020-12

**Authors:** Benuel Nyagaka, Stanslaus Kiilu Musyoki, Lucy Karani, Anthony Kebira Nyamache

**Affiliations:** 1 School of health sciences, Kisii University, P.O Box 408-40200, Kisii, Kenya; 2 Department of Microbiology, School of Pure and Applied Sciences, Kenyatta University, P.O. Box 43844 (00100), Nairobi, Kenya

**Keywords:** HIV infected elderly patients, Kisii Teaching and Referral Hospital, Kenya

## Abstract

**Background:**

A better understanding of the baseline characteristics of elderly people living with HIV/AIDS (PLWHA) is relevant because the world's HIV population is ageing.

**Objectives:**

This study aimed to evaluate the baseline characteristics of PLWHA aged ≥ 50years at recruitment to HIV/AIDS clinic compared against the viral load (VL) and CD4 count among patients attending Kisii Teaching and Referral Hospital (KTRH), Kenya.

**Methods:**

We retrospectively evaluated temporal inclinations of CD4 levels, viral load change and baseline demographic characteristics in the electronic records at the hospital using a mixed error-component model for 1329 PLWHA attending clinic between January 2008 and December 2019.

**Results:**

Findings showed a significant difference in the comparison between baseline VL and WHO AIDS staging (p=0.026). Overall VL levels decreased over the period significantly by WHO AIDS staging (p<0.0001). Significant difference was observed by gender (p<0.0001), across age groups (p<0.0001) and baseline CD4 counts (p=0.003). There were significant differences in WHO staging by CD4 count >200cell/mm3 (p=0.048) and residence (p=0.001).

**Conclusion:**

Age, WHO AIDS staging, gender and residence are relevant parameters associated with viral load decline and CD4 count in elderly PLWHA. A noticeable VL suppression was attained confirming possible attainment of VL suppression among PLWHA under clinical care.

## Introduction

Administration of antiretroviral therapy (ART) among human immunodeficiency virus (HIV) infected patients has led to increased survival rates among PLWHA.^1^ It is projected that by 2040, the number of PLWHA aged ≥50years will have grown by nearly three times in Sub-Saharan Africa (SSA) from an estimated 3.1 million in 2011 to 9.1 million^2^. With increasing access to ART across populations, there will be a need for long-term ART care.^3^,^4^ Previous studies from developed countries have shown distinct characteristics at diagnosis and clinical outcomes among PLWHA aged ≥50years compared to younger PLWHA.^5^

There are few research findings in SSA on the indicative features of HIV infection in PLWHA aged ≥50years receiving ART.^6^,^7^,^8^ Additionally, the restricted number studies that portray baseline indicative features, immunological response and mortality in elderly PLWHA have a limitation of small sample sizes.^9^,^10^ On account of this immediate information gap, furnishing supplementary data on the baseline indicative features of HIV infection among elderly PLWHA in sub-Saharan Africa is necessary. This study reports on the baseline characteristics of elderly PLWHA in Kenya which has not been addressed by previous studies. The purpose of our investigation was to examine baseline VL and CD4 count among people aged ≥50 years at the time of recruitment and identify disparities, if any, by gender, age group, current residence, WHO staging, patient source, and marital status in patients attending HIV/AIDS clinic at KTRH.

## Materials and Methods

### Study Area

The study was conducted at HIV care clinics of KTRH.

### Study Design and Data Collection

This study was a cross-sectional retrospective study using secondary electronic data obtained from HIV patients visiting an HIV/AIDS clinic at KTRH. Data on baseline CD4 count, baseline VL, last reported VL, WHO status, age, gender, marital status, the point at which individual patient source and residence was collected retrospectively from the electronic data maintained at from at KTRH.

### Demographics

Age was categorised as 50–59/60–69/70–79/≥80 years. Age was determined based on the age of the individual at the time of enrolment to the clinic. The current residence was categorised as urban or rural as previously defined.^11^ Gender was categorised as male or female while marital status was married/cohabiting, divorced or widowed. Patient Source was categorised as an outpatient, Prevention of mother-to-child transmission (PMTCT), Tuberculosis (TB), inpatient or Voluntary Counselling and Testing (VCT) clinics while WHO AIDS Staging was stage I/II/III/IV.

CD4 count and viral load: The baseline CD4 cell counts were placed in four categories as previously described,^12^,^13^,^14^ while VLs were classified into two guided by the previous related study.^15^ All undetectable VL values reported during the study period were replaced with 200 copies/mL following the CDC guideline.^16^

### Ethical considerations

University of Eastern Africa, Baraton research ethics committee, Ministry of Health, Ministry of Education and National Commission for Science, Technology and Innovation of Kenya approved this study.

## Statistical analysis

Descriptive statistics were used to examine baseline CD4 count, VL and demographic characteristics. Continuous measures were compared using the Kruskal-Wallis. Wilcoxon signed-rank test was performed to compare first and last, VL counts by demographics. The p-values ≤0.05 were considered statistically significant. All analyses were performed using SAS version 9.3.

## Results

Baseline CD4 counts, VL measurements and demographic characteristics of the study population were summarised (Table 1).

**Table 1:** Demographic Characteristics, CD4 Count and HIV Viral Load of PLWHA

Characteristics	Frequency (%)
**Gender**	
Male	547(41.2)
Female	782(58.8)
**Age group**	
50–59	934(70.3)
60–69	332(25.0)
70–79	60(4.5)
≥80	3(0.2)
**Marital Status**	
Married/cohabiting	1001(75.3)
Divorced	96(7.2)
Widowed	232(17.5)
**Residence**	
Urban	562(42.3)
Rural	767(57.7)
**Patient Source**	
Outpatient	1057(79.5)
PMTCT	62(4.7)
TB clinic	70(5.3)
Inpatient	61(4.6)
VCT	79(5.9)
**WHO Staging**	
I	556(41.8)
II	455(34.2)
III	295(22.2)
IV	23(1.7)
**CD4 count**	
≤200cells/mm^3^	320(24.1)
201–350cells/mm^3^	243(18.3)
351–500cells/mm^3^	399(30.0)
>500cells/mm^3^	367(27.6)
**Baseline VL**	
≤10000copies/mL	1244(93.6)
>10000copies/mL	85(6.4)

First CD4 and VL counts were defined as the first available value at the database.

Comparison of baseline characteristics by gender was determined (Table 2). Statistically significant differences were found in gender and median age (p<0.0001) and in gender and age groups (P<0.0001). Statistically significant differences were found in gender and CD4 categories (p=0.003).

**Table 2:** Comparisons Of Baseline Characteristics By Gender

Variable	Overall (*N=1329*)	Male (*N=547*)	Female (*N=782*)	*P-Value*
Mean Age(IQR)	57(50–89)	58(50–89)	56(50–83)	**<0.001**
**Age group**				
50–59	934	339	595	**<0.001**
60–69	332	173	159	
70–79	60	34	26	
≥80	3	1	2	
**Marital Status**				
Married/cohabiting	1001	413	588	0.433
Divorced	96	34	62	
Widowed	232	100	132	
**Residence**				
Urban	562	221	341	0.134
Rural	767	326	441	
**Patient Source**				
Outpatient	1057	431	626	0.973
PMTCT	62	27	35	
TB clinic	70	31	39	
Inpatient	61	25	36	
VCT	79	33	46	
**WHO staging**				
I	556	239	317	0.126
II	455	195	260	
III	295	106	189	
IV	23	7	16	
**Baseline CD4 count**				
≤200cells/mm^3^	320	158	162	**0.003**
201–350cells/mm^3^	243	98	145	
351–500cells/mm^3^	399	160	239	
>500cells/mm^3^	367	131	236	
**Baseline VL**				
≤10000copies/mL	1244	510	734	0.363
>10000copies/mL	85	37	48	

The p-value represents the comparison of the population baseline characteristics by gender.

Comparison of baseline characteristics by baseline CD4 cell count was determined (Table 3). Statistically significant differences were found in baseline CD4 count and WHO staging (P=0.048). Statistically significant differences were also found in baseline CD4 count by gender (P=0.003).

**Table 3:** Comparison Of Baseline Characteristics By CD4 Count

Variable	Mean CD4 count(cell/mm3) categories					*P-Value*

	Total (*N=1329*)	≤200 (*N=320*)	201–350 (*N=243*)	351–500 (*N=399*)	>500 (*N=367*)	
**Age Groups**						
50–59	934	211	186	284	253	0.152
60–69	332	93	50	99	90	
70–79	60	16	7	15	22	
≥80	3	0	0	1	2	
**Marital Status**						
Married/cohabiting	1001	251	177	297	276	0.524
Divorced	96	22	18	25	31	
Widowed	232	47	48	77	60	
**Residence**						
Urban	562	124	100	187	151	0.145
Rural	767	196	143	212	216	
**Patient Source**						
Outpatient	1057	251	198	321	287	0.832
PMTCT	62	14	13	15	20	
TB clinic	70	16	8	25	21	
Inpatient	61	15	13	16	17	
VCT	79	24	11	22	22	
**WHO staging**						
I	556	135	79	188	154	**0.048**
II	455	112	101	113	129	
III	295	67	59	90	79	
IV	23	6	4	8	5	
**Baseline viral** **load**						
≤10000copies/ml	1244	299	232	371	342	0.616
>10000copies/ml	85	21	11	28	25	
**Gender**						
Male	547	158	98	160	131	**0.003**
Female	782	162	145	239	236	

P-value represents the comparison of the population baseline characteristics by CD4 count

Comparison of baseline characteristics by WHO AIDS Staging was determined (Table 4). Statistically significant differences were found in WHO staging by baseline CD4 count categories (P=0.048). Statistically significant differences were found in WHO staging by residence (P=0.001) and WHO staging by baseline VL (P=0 .026).

**Table 4:** Comparison of Baseline Characteristics by WHO AIDS Staging

Variable	WHO AIDS Staging					*P-Value*

	Total (N=1329)	I (N=556)	II (N=455)	III (N=295)	IV (N=23)	
**Age Groups**						
50–59	934	388	312	221	13	0.284
60–69	332	142	119	61	10	
70–79	60	24	24	12	0	
≥80	3	2	0	1	0	
**Marital Status**						
Married/cohabiting	1001	419	335	229	18	0.649
Divorced	96	42	34	17	3	
Widowed	232	95	86	49	2	
**Residence**						
Urban	562	268	170	112	12	**0.001**
Rural	767	288	285	183	11	
**Patient Source**						
Outpatient	1057	439	362	238	18	0.944
PMTCT	62	28	18	15	1	
TB clinic	70	27	28	13	2	
Inpatient	61	25	21	15	0	
VCT	79	37	26	14	2	
**CD4 count**						
≤200	320	135	112	67	6	**0.048**
201–350	243	79	101	59	4	
351–500	399	188	113	90	8	
>500	367	154	129	79	5	
**Baseline VL**						
≤10000copies/mL	1244	521	428	277	18	**0.026**
>10000copies/mL	85	35	27	18	5	
**Gender**						
Male	547	239	195	106	7	0.126
Female	782	317	260	189	16	

The p-value represents the comparison of populations by WHO AIDS staging.

Comparison of the baseline characteristics by VL change was determined (Table 5). Statistically significant differences in changes in VL and were found by WHO staging (p<0.0001).

**Table 5:** Comparison Of Baseline Characteristics by Change in Viral Load

Variables	Mean Viral load(copies/mL)			

	Baseline	Last	Difference	*p-Value*
**Gender**				
Male	6588.1335	71.7788	6530.3333	0.455
Female	5092.1228	50.0358	5032.9182	
**Age Group**				
50–59	4473.3073	51.0418	4426.8746	0.275
60–69	8927.2440	83.0060	8826.3705	
70–79	7389.6833	50.2167	7339.4667	
≥80	151.3333	49.0000	102.3333	
**Marital Status**				
Married/Cohabiting	5358.9670	62.2338	5295.8430	0.637
Divorced	8978.2292	49.0000	8930.3750	
Widowed	5707.8623	58.9850	5810.9698	
**Residence**				
Urban	5676.8577	49.1174	5637.8788	0.993
Rural	5707.8623	58.9850	5656.3937	
**Patient Source**				
Outpatient	6226.5043	61.5109	6159.1599	0.704
PMTCT	365.3387	49.0000	316.2097	
TB clinic	4151.4143	49.2714	4102.4143	
Inpatient	2766.1475	49.0000	2717.1639	
VCT	6612.0127	49.3418	6648.0897	
**WHO AIDS**				
**Staging**				
I	4582.6241	51.4209	4530.3309	**<0.001**
II	5024.2220	73.9187	4937.3824	
III	5527.4814	50.8576	5496.1769	
IV	48747.0435	50.6522	48698.0435	
**CD4 count**				
≤200	7405.5281	53.9969	7349.7531	0.446
201–350	3761.1564	49.4897	3687.4239	
351–500	6997.6967	77.2005	6937.9724	
>500	4114.2725	49.8174	4065.4659	

## Discussion

We report baseline demographic characteristics, CD4 counts and VL for PLWHA aged ≥ 50years first-time HIV testers who were ART-naive and diagnosed with HIV. Majority of the PLWHA were originally tested at the outpatient clinic. The explanation could be that most people are unwilling to test for HIV and only get a reason to test when visiting health centres where they are recommended to take HIV test and the out-patient clinic is visited by most patients who seek treatment.

Overall, the HIV epidemic among the elderly who visited KTRH was dominated by adults aged 50–59years whose proportion was higher when compared to the national proportions^17^ for the same age group. The proportion of females was also higher compared to males which do not mimic the national inclination where the proportion of males is higher than females.^17^ This observation is because treatment guidelines have changed to emphasise early diagnosis, treatment, and attachment to care with a result that more individuals are receiving ART.^12^ Findings also show that the highest poportion of PLWHA aged ≥ 50years attending HIV/AIDS clinic are married/cohabiting. A feasible explanation is that by age of 24.8 years most Kenyans are married.^18^

In the stated study period VL declined. Among the reasons for this observation is that the current ART regimens have been improved and are more comfortably endured. It is also plausible that patients with higher VL die earlier and the people who remain have lower VL.^12^ We observed a higher decline in VL among PL-WHA in WHO AIDS stages III/IV as compared to WHO AIDS stages I/II. Probably this is because many individuals with WHO AIDS stages I/II may have entered the study with lower VLs compared to those at stage III/IV making it difficult to detect any additional VL decline in this group. There were significantly more individuals whose baseline VL was ≤10000copies/mL at recruitment just as there were more individuals in WHO AIDS stages I/II. This again can be explained by a change in treatment guidelines as explained above. When we adjusted VL for age group and WHO AIDS staging, we observed fewer individuals in the age groups 70 years and above. This could be explained by natural attrition with an increase in age. Life expectancy in Kenya is 66.7 years. 19

There was a significantly higher number of females in each CD4 category which was not clear. Notwithstanding some studies suggest that the probability of late testing for HIV is higher for men compared to females.^20^,^21^ Higher number of individuals in WHO AIDS stages I/II/III was observed to have rural residence. It is plausible that most people aged ≥ 50years could have retired and relocated to rural homes at least for the Kenyan case.

There were some limitations in our study. Data was not available for PLWHA who dropped out of care and those who were not tested. Our study did not include co-morbidities which impinge HIV treatment particularly in PLWHA aged ≥ 50years.^22^

## Conclusion and recommendations

This study uncovered unavoidable indicative features (Age, WHO AIDS stages, Gender and Residence) as associated factors to CD4 count and VL decline. Therefore CD4 count, VL, age, WHO AIDS stages, gender and residence should be utilised in solving health care challenges associated with elderly PLWHA. Additionally, Noticeable VL suppression was attained during the study period confirming possible attainment of VL suppression among elderly PLWHA under clnical care.

## References

[1] Autenrieth CS, Beck EJ, Stelzle D, Mallouris C, Mahy M, Ghys P (2018). Global and Regional Trends of People Living with HIV Aged 50 and over : Estimates and Projections for 2000–2020. PLoS One.

[2] Kharsany AB, Karim QA (2016). HIV Infection and AIDS in sub-Saharan Africa: Current Status, Challenges and Opportunities. The Open AIDS Journal.

[3] Kiplagat J, Mwangi A, Keter A (2018). Retention in Care among Older Adults Living with HIV in Western Kenya: A Retrospective Observational Cohort Study. PLoS One.

[4] Nansseu JRN, Jean JRB (2017). Antiretroviral Therapy Related Adverse Effects: Can Sub-Saharan Africa Cope with the New ‘Test and Treat’ Policy of the World Health Organization?. Infectious Diseases of Poverty.

[5] Tweya H, Feldacker C, Heller T (2017). Characteristics and Outcomes of Older HIV-Infected Patients Receiving Antiretroviral Therapy in Malawi: A Retrospective Observation Cohort Study. PLoS One.

[6] Negin J, Martiniuk A, Cumming RG (2012). Prevalence of HIV and Chronic Comorbidities among Older Adults. AIDS.

[7] Martin CP, Fain MJ, Klotz SA (2008). The Older HIV-Positive Adult: A Critical Review of the Medical Literature. American Journal of Medicine.

[8] Aboderin IAG, Beard JR (2015). Older People's Health in Sub-Saharan Africa. The Lancet.

[9] Semeere AS, Lwanga I, Sempa J (2014). Mortality andImmunological Recovery among Older Adults on Antiretroviral Therapy at a Large Urban HIV Clinic in Kampala, Uganda. Journal of Acquired Immune Deficiency Syndromes.

[10] Maskew M, Brennan AT, MacPhail AP, Sanne IM, Fox MP (2012). Poorer ART Outcomes with Increasing Age at a Large Public Sector HIV Clinic in Johannesburg, South Africa. Journal of the International Association of Physicians in AIDS Care.

[11] United Nations, Department of Economic and Social Affairs, Population Division (2019). World Urbanization Prospects: The 2018 Revision (ST/ESA/SER.A/420).

[12] Chakraborty H, Medha I, Wayne AD, Ashok VS, Helmut A, Aron W (2015). Disparities in Viral Load and CD4 Count Trends among HIV-Infected Adults in South Carolina. AIDS Patient Care and STDs.

[13] Li X, Margolick J, Jamieson B, Rinaldo C, Phair J, Jacobson L (2011). CD4++ T-Cell Counts and Plasma HIV-1 RNA Levels beyond 5 Years of Highly Active Antiretroviral Therapy. Journal of Acquired Immune Deficiency Syndromes.

[14] Pereira MF, Luz E, Netto EM, Barbosa MHF, Brites C (2018). Low Variation in Initial CD4 Cell Count in a HIV Referral Center, in Salvador, Brazil, from 2002 to 2015. Brazilian Journal of Infectious Diseases.

[15] Murnane PM, Hughes JP, Celum C (2012). Using Plasma Viral Load to Guide Antiretroviral Therapy Initiation to Prevent HIV-1 Transmission. PLoS One.

[16] Centers for Disease Control and Prevention (2011). Guidance on Community Viral Load: A Family of Measures, Definitions, and Method for Calculation.

[17] National AIDS and STI Control Programme (NASCOP), Kenya (2014). Kenya AIDS Indicator Survey 2012: Final Report.

[18] United Nations, Department of Economic and Social Affairs, Population Division (2014). World Fertility Report 2013: Fertility at the Extremes (United Nations publication).

[19] United Nations, Department of Economic and Social Affairs, Population Division (2019). World Mortality 2019: Data Booklet (ST/ ESA/SER.A/436).

[20] Hall HI, Frazier EL, Rhodes P (2013). Differences in Human Immunodeficiency Virus Care and Treatment among Subpopulations in the United States. JAMA Internal Medicine.

[21] Muthulingam D, Chin J, Hsu L, Scheer S, Schwarcz S (2013). Disparities in Engagement in Care and Viral Suppression among Persons with HIV. Journal of Acquired Immune Deficiency Syndromes.

[22] Warren-Jeanpiere L, Dillaway H, Hamilton P, Young M, Goparaju L (2014). Taking It One Day at a Time: African American Women Aging with Hiv and Co-Morbidities. AIDS Patient Care and STDs.

